# Sir James Crichton-Browne on Adulteration

**Published:** 1907-09-28

**Authors:** 


### Sir James Crichton-Browne on Adulteration.

Sir James Crichton-Browne has figured as a champion on many controversial fields, and his presence as a combatant invariably insures both a vigorous and an exciting contest. There is no lack of energy in his strokes, and even those who feel their weight need not withhold admiration for the skill with which they are delivered. To change the figure, we may venture to say that Sir James is a master of the art of thrilling and picturesque denunciation, and his sonorous phrases merit at least the success due to efficient artistic effort. Possibly a suspicion may sometimes arise that the rhetoric is a trifle gaudy and that an ambition to make the flesh creep is not wholly absent. Yet the orator commands, as we are sure he deserves, attention, and even for his most thrilling flights he usually manages to suggest the existence of a substantial justification of fact. Certain it is that his reforming efforts merit every sympathy from the members of the profession, of which he is a distinguished ornament, and who have for their care and responsibility the health of the nation. We therefore turn with interest, guarded perhaps with some degree of circumspection, to Sir James's presidential address to the Association of Sanitary Inspectors.

In the address in question there is presented an extremely gloomy and depressing picture of the condition of our food supplies and of the dangers which these involve both to the individual and to the public health. The methods of corruption, we are told, are becoming more subtle, and substitution, if not a fine art, is rapidly attaining to the level of an exact science. Adulteration and manipulation prevail on a large scale, and their range, so far from diminishing, is extending. Taking individual instances, the practice of reducing the quality of milk by adding water and abstracting cream is becoming more common; the sophistication of butter is increasing, and, by the efforts of "ingenious chemists abroad," this is conducted so as successfully to defy detection; oysters and cockles are polluted with sewage; watercress may have typhoid fever on its leaves; tinned meats carry ptomaines; hams trichinosis; many of our wines are concocted without assistance from the juice of the grape; nuts of a poisonous description have been imported; and even the medicines we give the sick are sometimes deprived of their healing power. To these must be added the risk of tuberculosis both from milk and meat, which, under existing arrangements, are not subjected to the protective influence of adequate and skilful inspection. All this forms, it must be admitted, a most melancholy and unhappy catalogue. Nevertheless, though we do not in the least propose to question the accuracy of Sir James's statements, they suggest one or two remarks which may help us at least to comparative courage.

Accepting, then, the position as presented, it may be asked whether we must come to the conclusion that things are as bad now as they were, say, fifty years ago. For what Sir James Crichton-Browne says to-day, is very much what the poet sang in the middle of the nineteenth century:—

> "And chalk and alum and plaster are sold to the poor for bread,
> And the spirit of murder lurks in the very means of life.
>
> . . . . . . . .
>
> While another is cheating the sick of a few last gasps, as he sits
> To pestle a poison'd poison behind his crimson lights."

The two pictures have a close family resemblance, and if the inference just suggested must be admitted, things are indeed in a parlous condition. And the seriousness of the position is made manifest when it is remembered that in the interval between the two descriptions social reformers of all varieties have been at work, Acts of Parliament have been put into operation, and numerous expert analysts and inspectors have been on the look-out to detect the careless or wilful adulterator of food. All this has cost, and is still costing, large sums of money, and we fear that practical men of the world, on reading Sir James's address, will come to the conclusion that this money has been largely spent in vain. They will probably also proceed to argue that if the existing machinery has proved a failure it is useless, and indeed worse than useless, to erect more of the same nature. Inspectors increased in number and provided with more elaborate training, will mean yet more expense to the community, and may it not be argued that even with this equipment we have no guarantee that the "skilful foreign chemist" will not be too much for us. In short, with all deference to Sir James Crichton-Browne, we suggest that the unrelieved gloom of his address is calculated to defeat the main object with which it was delivered. By all means let the practices of adulteration and substitution be dragged into the light of day. But let us at the same time hear of what has been successfully accomplished to restrain these practices, and do not force on the public mind the conviction that in spite of our existing sanitary regulations the criminal is winning all along the line. In addition, there comes a time, too, it must be remembered, when the conditions of insurance are so onerous that men will prefer rather to run the risk than to pay the premium.

